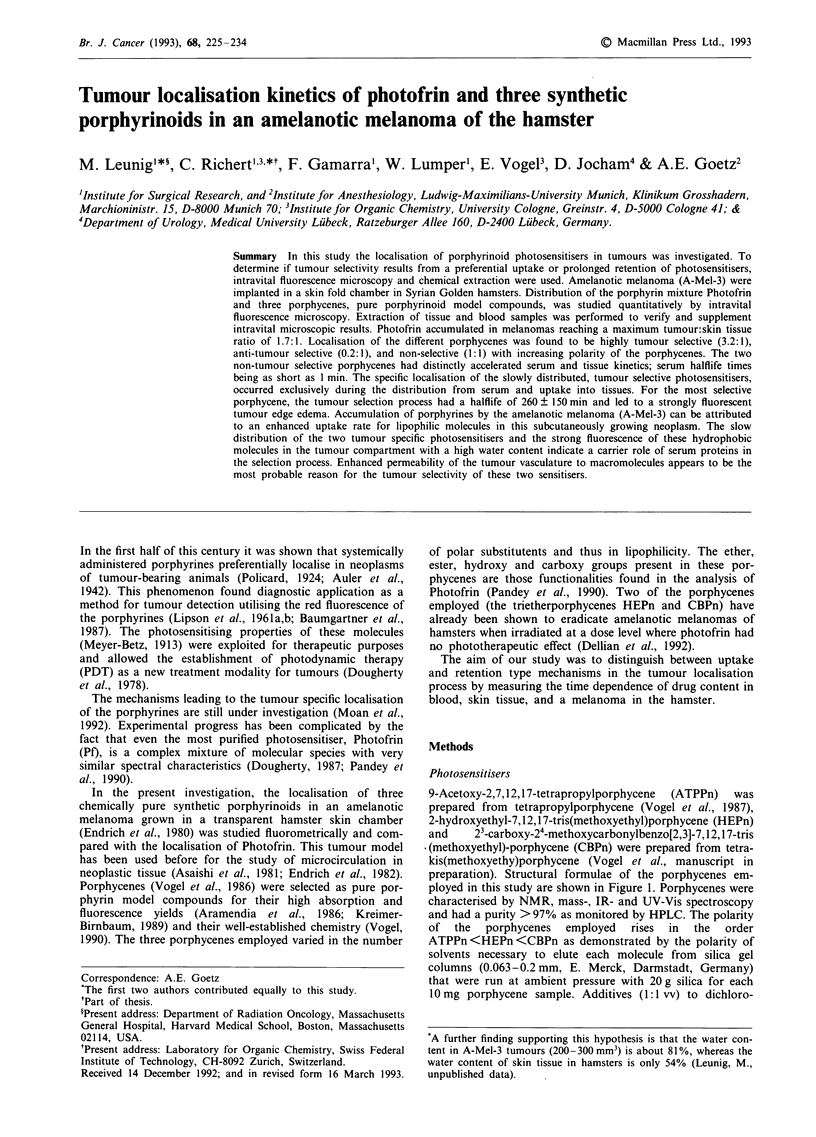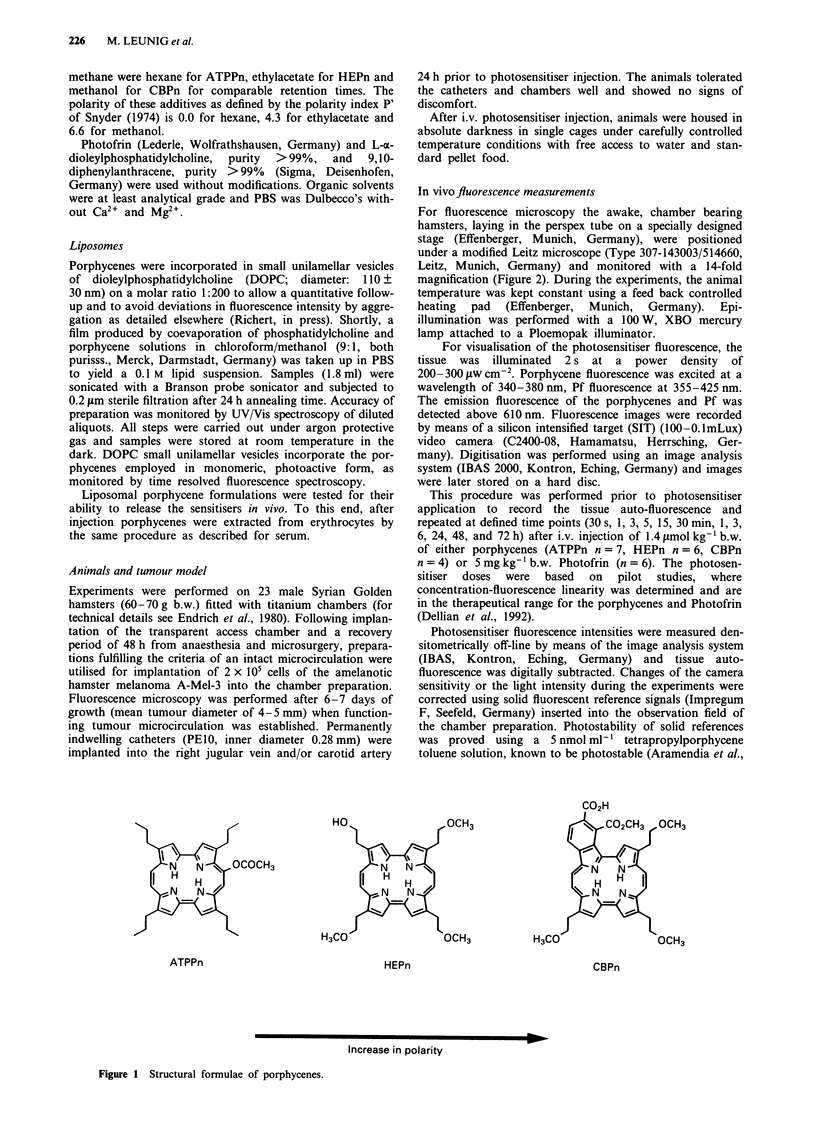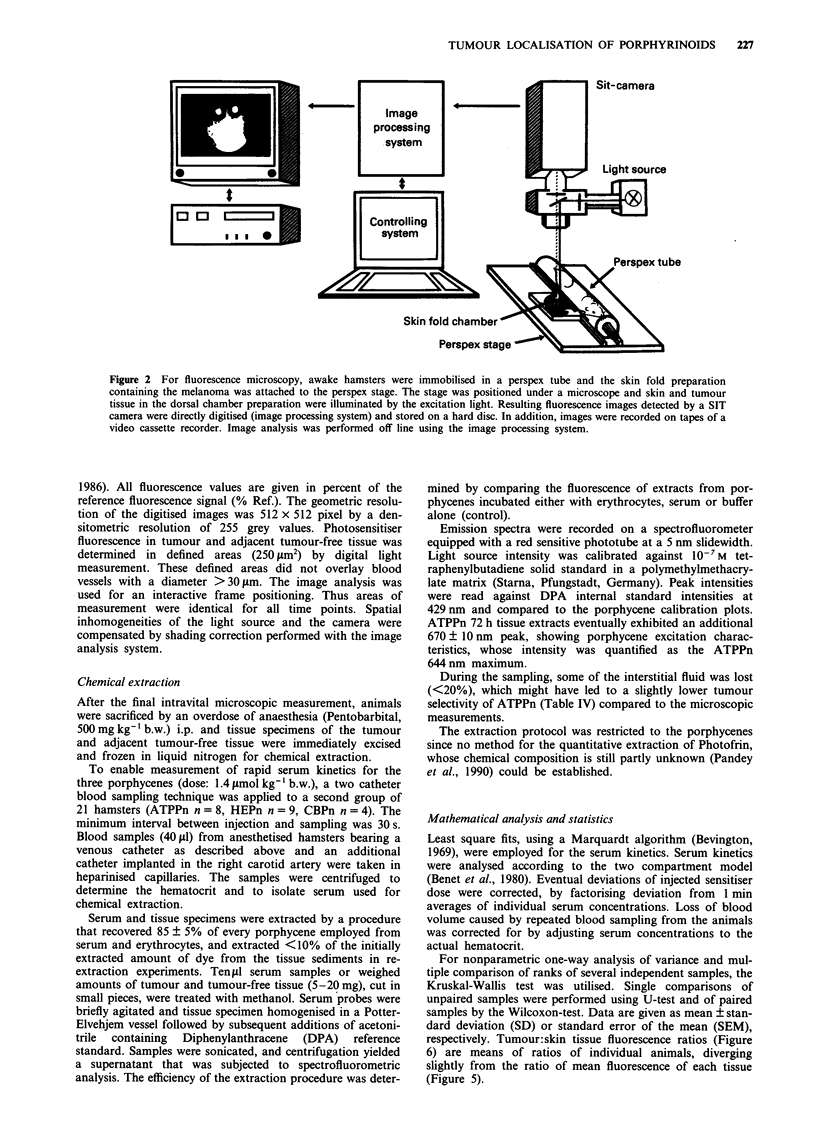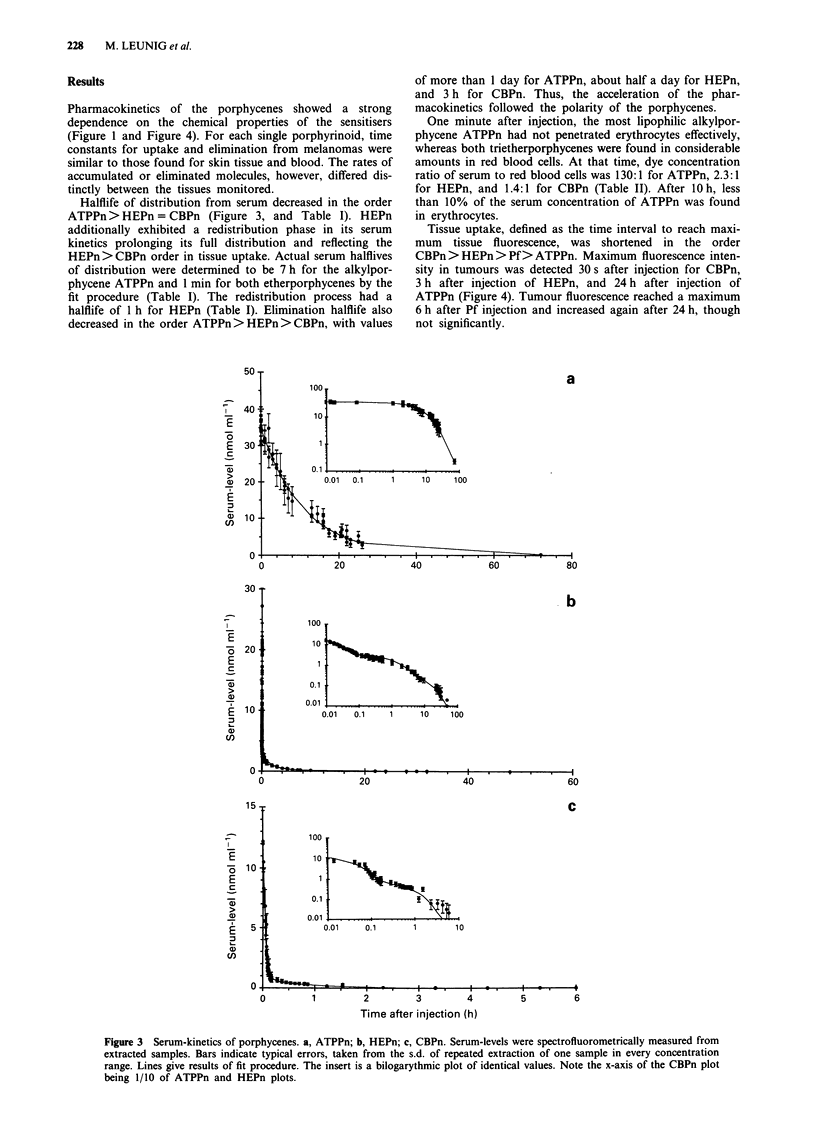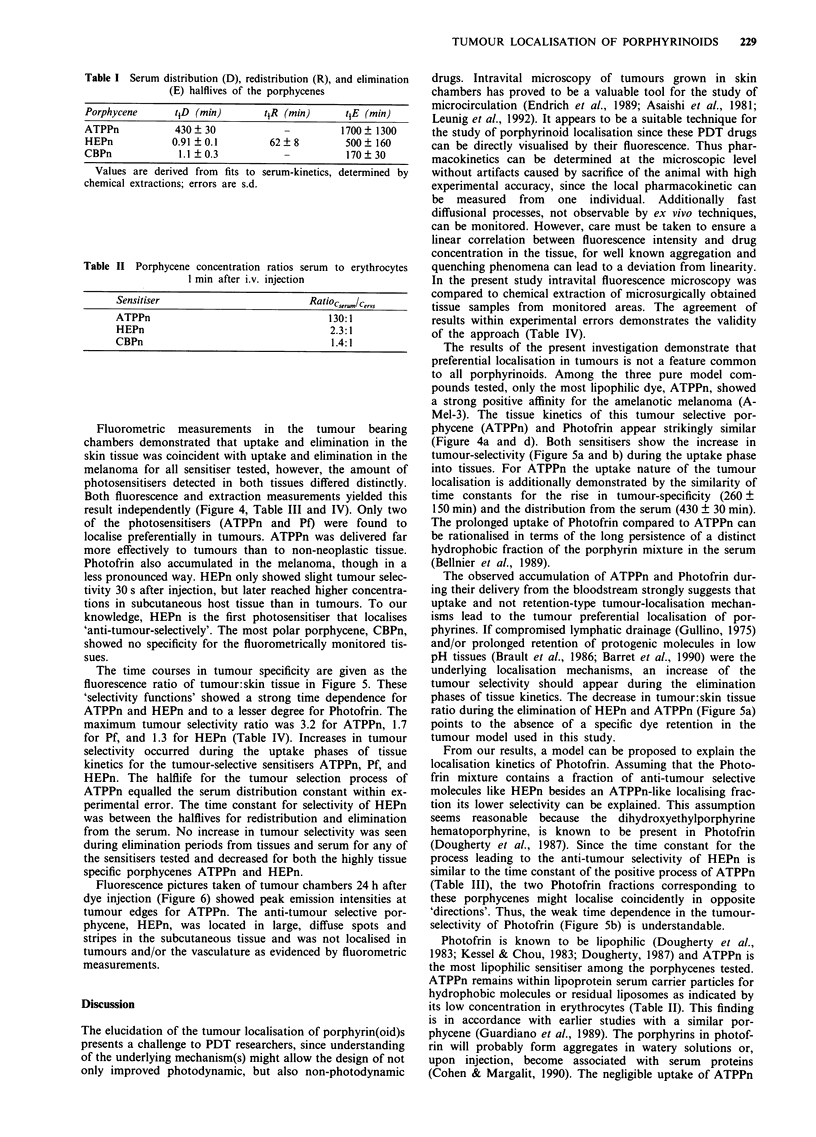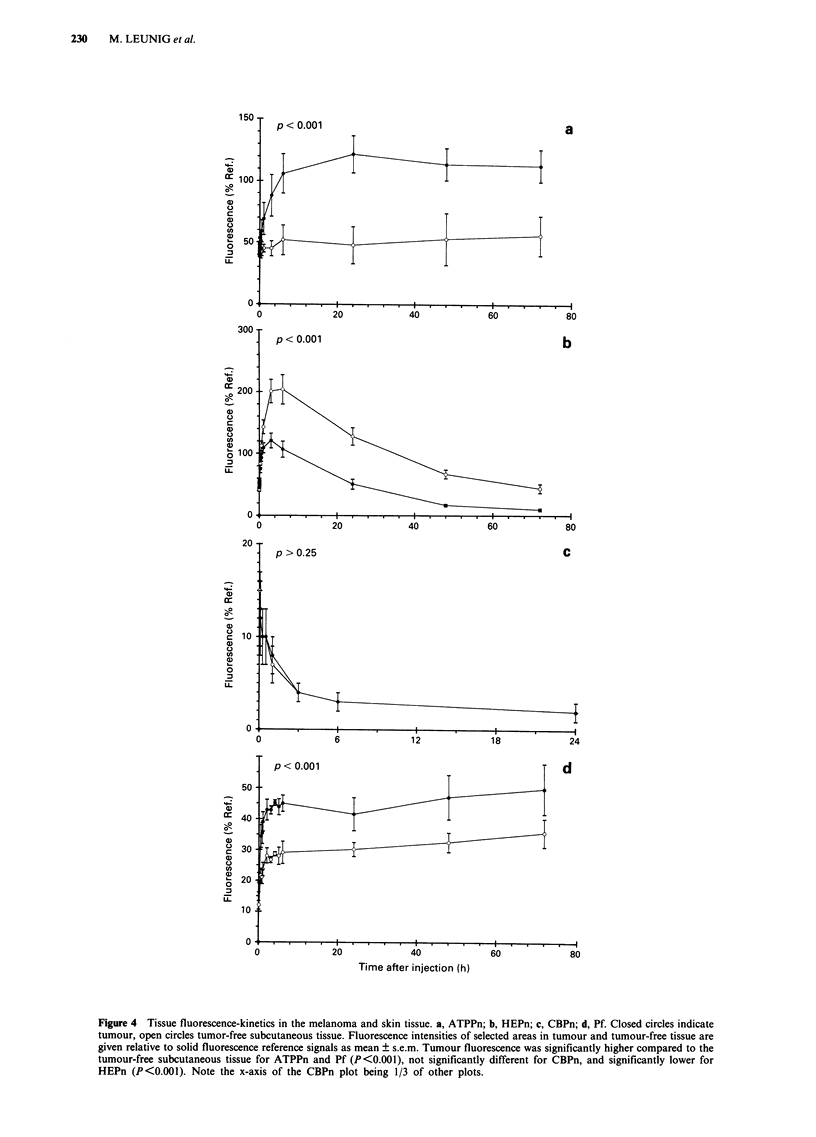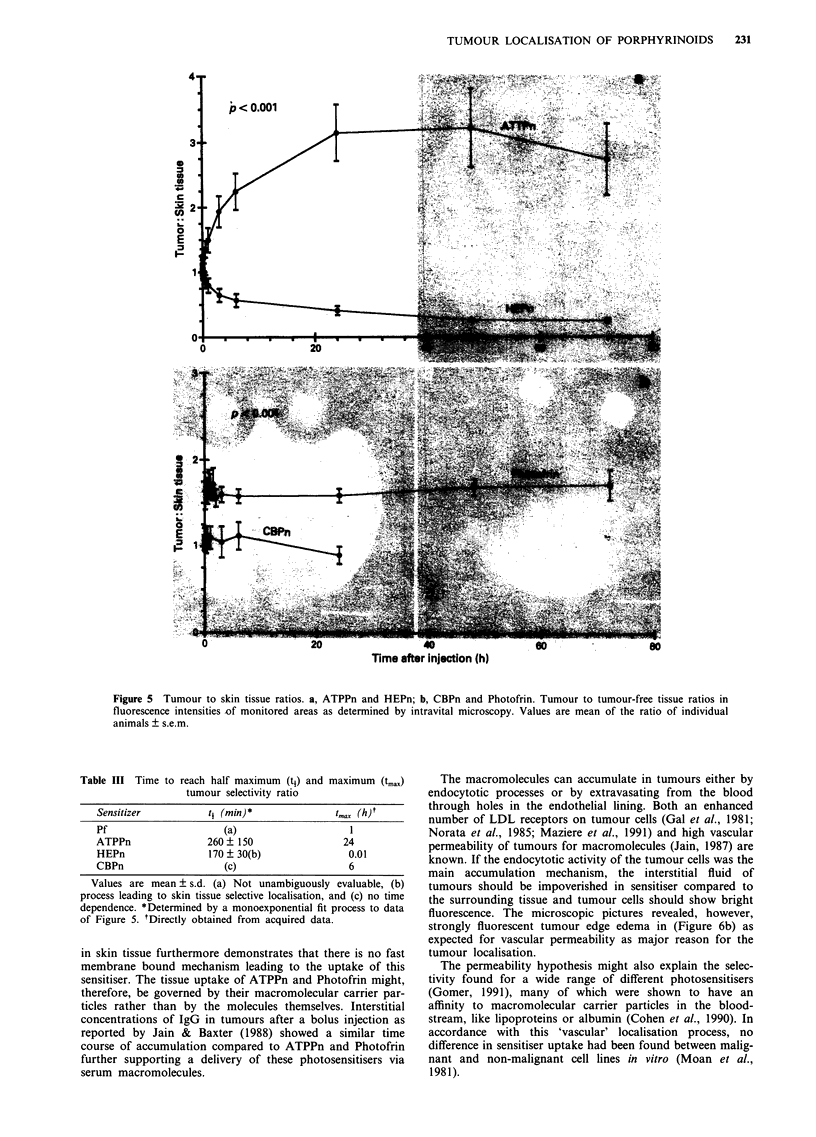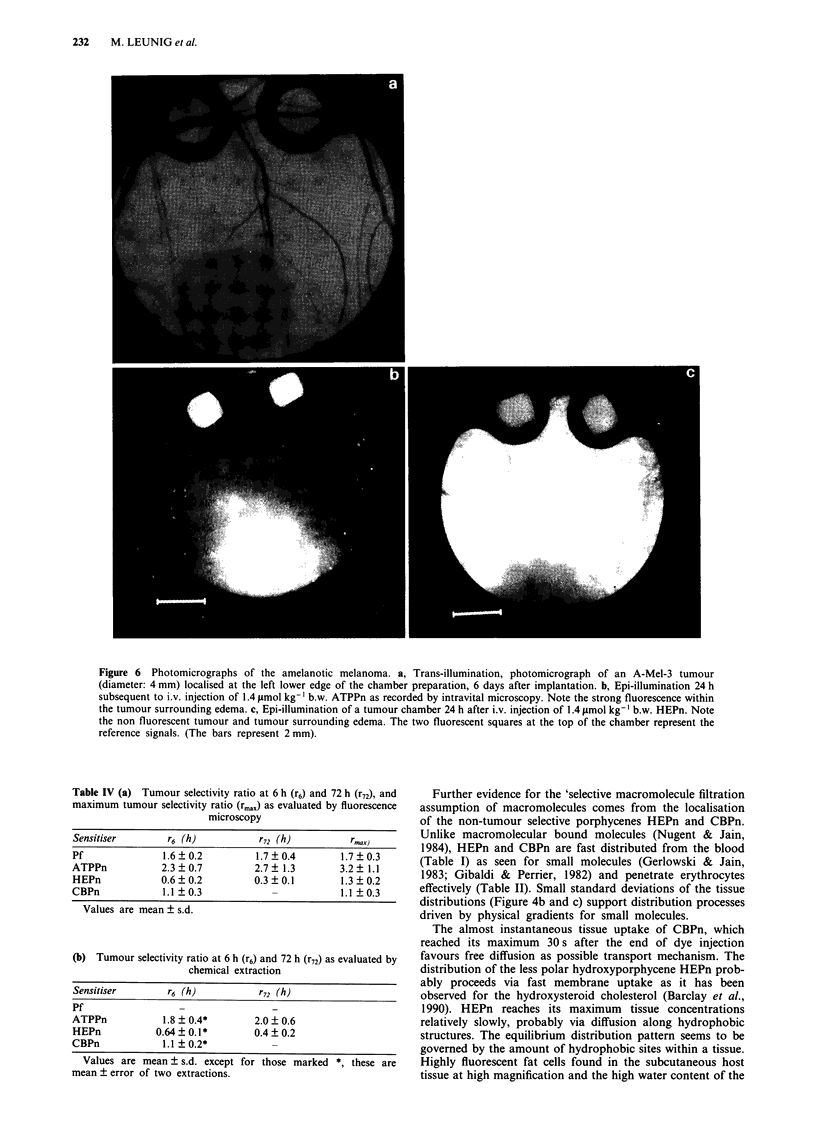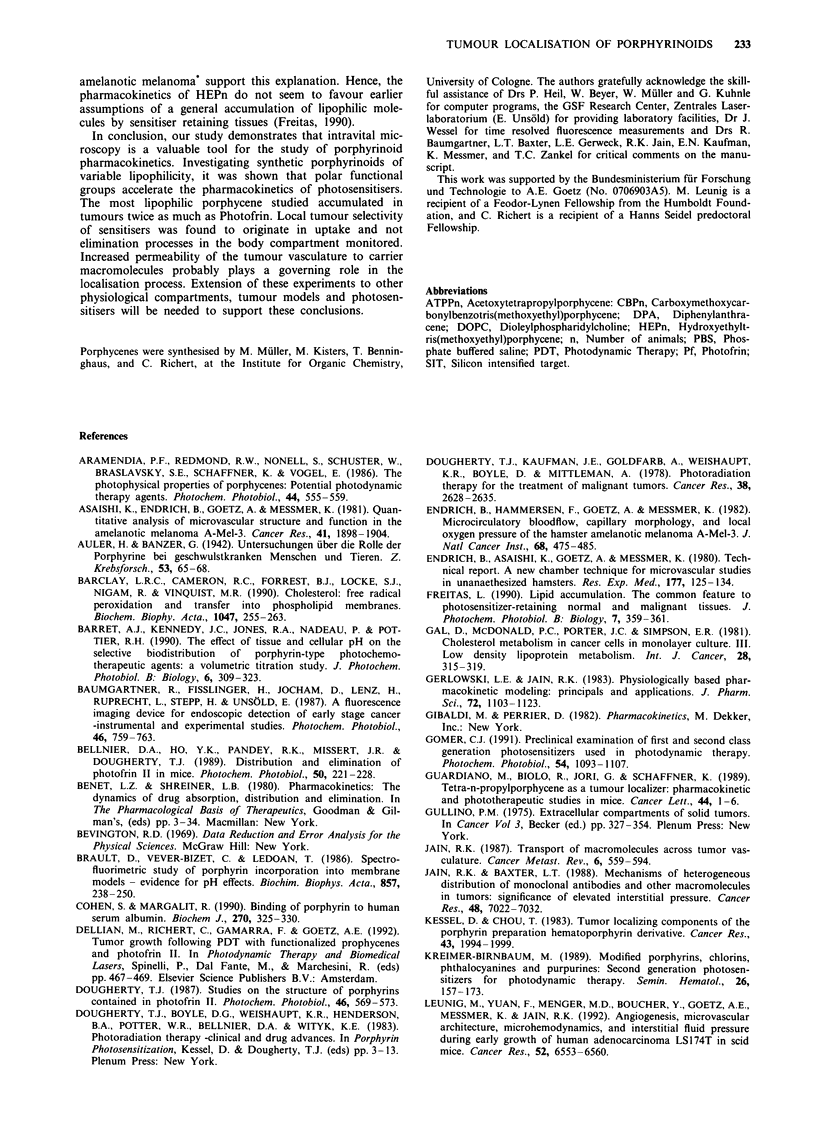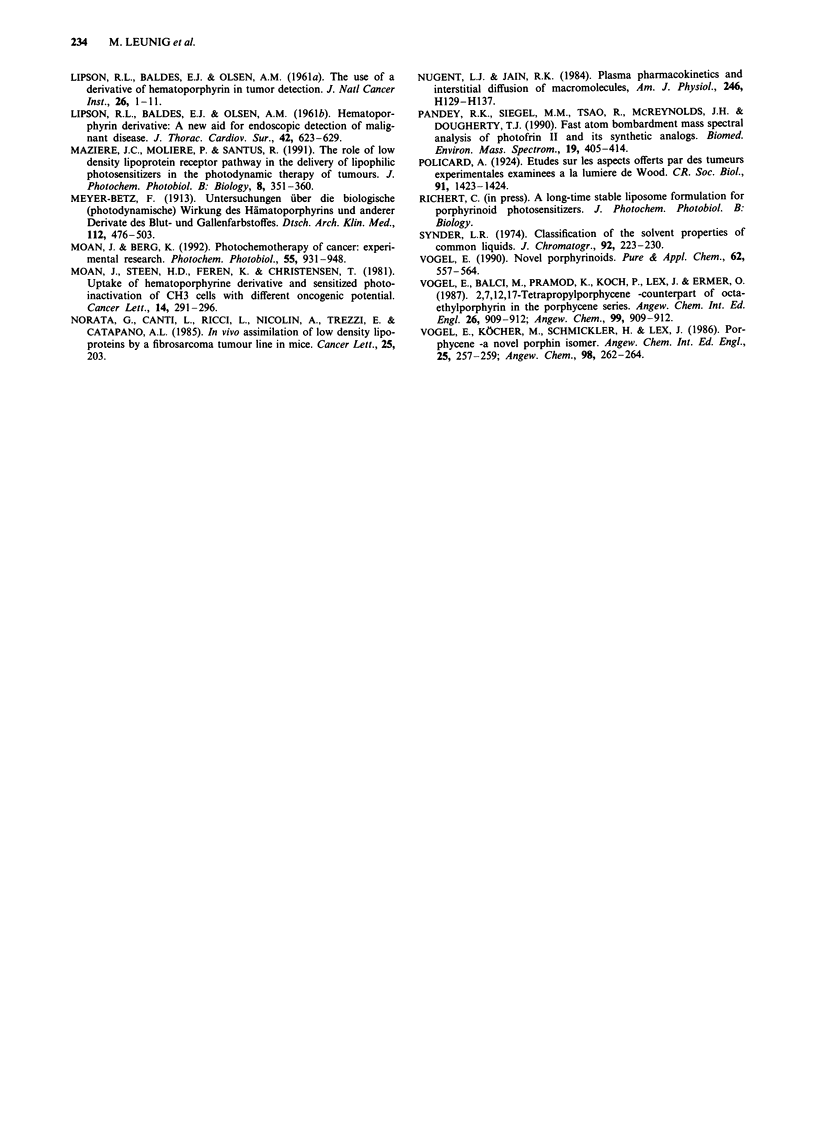# Tumour localisation kinetics of photofrin and three synthetic porphyrinoids in an amelanotic melanoma of the hamster.

**DOI:** 10.1038/bjc.1993.320

**Published:** 1993-08

**Authors:** M. Leunig, C. Richert, F. Gamarra, W. Lumper, E. Vogel, D. Jocham, A. E. Goetz

**Affiliations:** Institute for Surgical Research, Ludwig-Maximilians-University Munich, Klinikum Grosshadern, Munich, Germany.

## Abstract

**Images:**


Br. J. Cancer (1993), 68, 225–234 © Macmillan Press Ltd., 1993

### Tumour localisation kinetics of photofrin and three synthetic porphyrinoids in an amelanotic melanoma of the hamster

M. Leunig[1]*§, C. Richert[1,3,]*†, F. Gamarra[1], W. Lumper[1], E. Vogel[3], D. Jocham[4] & A.E. Goetz[2]

[1]*Institute for Surgical Research, and* [2]*Institute for Anesthesiology, Ludwig-Maximilians-University Munich, Klinikum Grosshadern, Marchioninistr. 15, D-8000 Munich 70;* [3]*Institute for Organic Chemistry, University Cologne, Greinstr. 4, D-5000 Cologne 41; &* [4]*Department of Urology, Medical University Lübeck, Ratzeburger Allee 160, D-2400 Lübeck, Germany.*

**Summary** In this study the localisation of porphyrinoid photosensitisers in tumours was investigated. To determine if tumour selectivity results from a preferential uptake or prolonged retention of photosensitisers, intravital fluorescence microscopy and chemical extraction were used. Amelanotic melanoma (A-Mel-3) were implanted in a skin fold chamber in Syrian Golden hamsters. Distribution of the porphyrin mixture Photofrin and three porphycenes, pure porphyrinoid model compounds, was studied quantitatively by intravital fluorescence microscopy. Extraction of tissue and blood samples was performed to verify and supplement intravital microscopic results. Photofrin accumulated in melanomas reaching a maximum tumour:skin tissue ratio of 1.7:1. Localisation of the different porphycenes was found to be highly tumour selective (3.2:1), anti-tumour selective (0.2:1), and non-selective (1:1) with increasing polarity of the porphycenes. The two non-tumour selective porphycenes had distinctly accelerated serum and tissue kinetics; serum halflife times being as short as 1 min. The specific localisation of the slowly distributed, tumour selective photosensitisers, occurred exclusively during the distribution from serum and uptake into tissues. For the most selective porphycene, the tumour selection process had a halflife of 260 ± 150 min and led to a strongly fluorescent tumour edge edema. Accumulation of porphyrines by the amelanotic melanoma (A-Mel-3) can be attributed to an enhanced uptake rate for lipophilic molecules in this subcutaneously growing neoplasm. The slow distribution of the two tumour specific photosensitisers and the strong fluorescence of these hydrophobic molecules in the tumour compartment with a high water content indicate a carrier role of serum proteins in the selection process. Enhanced permeability of the tumour vasculature to macromolecules appears to be the most probable reason for the tumour selectivity of these two sensitisers.

In the first half of this century it was shown that systemically administered porphyrines preferentially localise in neoplasms of tumour-bearing animals (Policard, 1924; Auler *et al.*, 1942). This phenomenon found diagnostic application as a method for tumour detection utilising the red fluorescence of the porphyrines (Lipson *et al.*, 1961a,b; Baumgartner *et al.*, 1987). The photosensitising properties of these molecules (Meyer-Betz, 1913) were exploited for therapeutic purposes and allowed the establishment of photodynamic therapy (PDT) as a new treatment modality for tumours (Dougherty *et al.*, 1978).

The mechanisms leading to the tumour specific localisation of the porphyrines are still under investigation (Moan *et al.*, 1992). Experimental progress has been complicated by the fact that even the most purified photosensitiser, Photofrin (Pf), is a complex mixture of molecular species with very similar spectral characteristics (Dougherty, 1987; Pandey *et al.*, 1990).

In the present investigation, the localisation of three chemically pure synthetic porphyrinoids in an amelanotic melanoma grown in a transparent hamster skin chamber (Endrich *et al.*, 1980) was studied fluorometrically and compared with the localisation of Photofrin. This tumour model has been used before for the study of microcirculation in neoplastic tissue (Asaishi *et al.*, 1981; Endrich *et al.*, 1982). Porphycenes (Vogel *et al.*, 1986) were selected as pure porphyrin model compounds for their high absorption and fluorescence yields (Aramendia *et al.*, 1986; Kreimer-Birnbaum, 1989) and their well-established chemistry (Vogel, 1990). The three porphycenes employed varied in the number of polar substitutents and thus in lipophilicity. The ether, ester, hydroxy and carboxy groups present in these porphycenes are those functionalities found in the analysis of Photofrin (Pandey *et al.*, 1990). Two of the porphycenes employed (the trietherporphycenes HEPn and CBPn) have already been shown to eradicate amelanotic melanomas of hamsters when irradiated at a dose level where photofrin had no phototherapeutic effect (Dellian *et al.*, 1992).

The aim of our study was to distinguish between uptake and retention type mechanisms in the tumour localisation process by measuring the time dependence of drug content in blood, skin tissue, and a melanoma in the hamster.

### Methods

### *Photosensitisers*

9-Acetoxy-2,7,12,17-tetrapropylporphycene (ATPPn) was prepared from tetrapropylporphycene (Vogel *et al.*, 1987), 2-hydroxyethyl-7,12,17-tris(methoxyethyl)porphycene (HEPn) and $2^3$-carboxy-$2^4$-methoxycarbonylbenzo[2,3]-7,12,17-tris (methoxyethyl)-porphycene (CBPn) were prepared from tetrakis(methoxyethy)porphycene (Vogel *et al.*, manuscript in preparation). Structural formulae of the porphycenes employed in this study are shown in Figure 1. Porphycenes were characterised by NMR, mass-, IR- and UV-Vis spectroscopy and had a purity >97% as monitored by HPLC. The polarity of the porphycenes employed rises in the order ATPPn<HEPn<CBPn as demonstrated by the polarity of solvents necessary to elute each molecule from silica gel columns (0.063–0.2 mm, E. Merck, Darmstadt, Germany) that were run at ambient pressure with 20 g silica for each 10 mg porphycene sample. Additives (1:1 vv) to dichloro-

Correspondence: A.E. Goetz
*The first two authors contributed equally to this study.
†Part of thesis.
§Present address: Department of Radiation Oncology, Massachusetts General Hospital, Harvard Medical School, Boston, Massachusetts 02114, USA.
†Present address: Laboratory for Organic Chemistry, Swiss Federal Institute of Technology, CH-8092 Zurich, Switzerland.
Received 14 December 1992; and in revised form 16 March 1993.

*A further finding supporting this hypothesis is that the water content in A-Mel-3 tumours (200–300 $mm^3$) is about 81%, whereas the water content of skin tissue in hamsters is only 54% (Leunig, M., unpublished data).

methane were hexane for ATPPn, ethylacetate for HEPn and methanol for CBPn for comparable retention times. The polarity of these additives as defined by the polarity index P' of Snyder (1974) is 0.0 for hexane, 4.3 for ethylacetate and 6.6 for methanol.

Photofrin (Lederle, Wolfrathshausen, Germany) and L-α-dioleylphosphatidylcholine, purity >99%, and 9,10-diphenylanthracene, purity >99% (Sigma, Deisenhofen, Germany) were used without modifications. Organic solvents were at least analytical grade and PBS was Dulbecco's without $Ca^{2+}$ and $Mg^{2+}$.

### Liposomes

Porphycenes were incorporated in small unilamellar vesicles of dioleylphosphatidylcholine (DOPC; diameter: 110 ± 30 nm) on a molar ratio 1:200 to allow a quantitative follow-up and to avoid deviations in fluorescence intensity by aggregation as detailed elsewhere (Richert, in press). Shortly, a film produced by coevaporation of phosphatidylcholine and porphycene solutions in chloroform/methanol (9:1, both purisss., Merck, Darmstadt, Germany) was taken up in PBS to yield a 0.1 M lipid suspension. Samples (1.8 ml) were sonicated with a Branson probe sonicator and subjected to 0.2 μm sterile filtration after 24 h annealing time. Accuracy of preparation was monitored by UV/Vis spectroscopy of diluted aliquots. All steps were carried out under argon protective gas and samples were stored at room temperature in the dark. DOPC small unilamellar vesicles incorporate the porphycenes employed in monomeric, photoactive form, as monitored by time resolved fluorescence spectroscopy.

Liposomal porphycene formulations were tested for their ability to release the sensitisers *in vivo*. To this end, after injection porphycenes were extracted from erythrocytes by the same procedure as described for serum.

### Animals and tumour model

Experiments were performed on 23 male Syrian Golden hamsters (60–70 g b.w.) fitted with titanium chambers (for technical details see Endrich *et al.*, 1980). Following implantation of the transparent access chamber and a recovery period of 48 h from anaesthesia and microsurgery, preparations fulfilling the criteria of an intact microcirculation were utilised for implantation of $2 \times 10^5$ cells of the amelanotic hamster melanoma A-Mel-3 into the chamber preparation. Fluorescence microscopy was performed after 6–7 days of growth (mean tumour diameter of 4–5 mm) when functioning tumour microcirculation was established. Permanently indwelling catheters (PE10, inner diameter 0.28 mm) were implanted into the right jugular vein and/or carotid artery 24 h prior to photosensitiser injection. The animals tolerated the catheters and chambers well and showed no signs of discomfort.

After i.v. photosensitiser injection, animals were housed in absolute darkness in single cages under carefully controlled temperature conditions with free access to water and standard pellet food.

### In vivo *fluorescence measurements*

For fluorescence microscopy the awake, chamber bearing hamsters, laying in the perspex tube on a specially designed stage (Effenberger, Munich, Germany), were positioned under a modified Leitz microscope (Type 307-143003/514660, Leitz, Munich, Germany) and monitored with a 14-fold magnification (Figure 2). During the experiments, the animal temperature was kept constant using a feed back controlled heating pad (Effenberger, Munich, Germany). Epi-illumination was performed with a 100 W, XBO mercury lamp attached to a Ploemopak illuminator.

For visualisation of the photosensitiser fluorescence, the tissue was illuminated 2 s at a power density of 200–300 $\mu W\,cm^{-2}$. Porphycene fluorescence was excited at a wavelength of 340–380 nm, Pf fluorescence at 355–425 nm. The emission fluorescence of the porphycenes and Pf was detected above 610 nm. Fluorescence images were recorded by means of a silicon intensified target (SIT) (100–0.1mLux) video camera (C2400-08, Hamamatsu, Herrsching, Germany). Digitisation was performed using an image analysis system (IBAS 2000, Kontron, Eching, Germany) and images were later stored on a hard disc.

This procedure was performed prior to photosensitiser application to record the tissue auto-fluorescence and repeated at defined time points (30 s, 1, 3, 5, 15, 30 min, 1, 3, 6, 24, 48, and 72 h) after i.v. injection of 1.4 $\mu mol\,kg^{-1}$ b.w. of either porphycenes (ATPPn $n = 7$, HEPn $n = 6$, CBPn $n = 4$) or 5 $mg\,kg^{-1}$ b.w. Photofrin ($n = 6$). The photosensitiser doses were based on pilot studies, where concentration-fluorescence linearity was determined and are in the therapeutical range for the porphycenes and Photofrin (Dellian *et al.*, 1992).

Photosensitiser fluorescence intensities were measured densitometrically off-line by means of the image analysis system (IBAS, Kontron, Eching, Germany) and tissue auto-fluorescence was digitally subtracted. Changes of the camera sensitivity or the light intensity during the experiments were corrected using solid fluorescent reference signals (Impregum F, Seefeld, Germany) inserted into the observation field of the chamber preparation. Photostability of solid references was proved using a 5 $nmol\,ml^{-1}$ tetrapropylporphycene toluene solution, known to be photostable (Aramendia *et al.*,

**Figure 1** Structural formulae of porphycenes.

**Figure 2** For fluorescence microscopy, awake hamsters were immobilised in a perspex tube and the skin fold preparation containing the melanoma was attached to the perspex stage. The stage was positioned under a microscope and skin and tumour tissue in the dorsal chamber preparation were illuminated by the excitation light. Resulting fluorescence images detected by a SIT camera were directly digitised (image processing system) and stored on a hard disc. In addition, images were recorded on tapes of a video cassette recorder. Image analysis was performed off line using the image processing system.

1986). All fluorescence values are given in percent of the reference fluorescence signal (% Ref.). The geometric resolution of the digitised images was 512 × 512 pixel by a densitometric resolution of 255 grey values. Photosensitiser fluorescence in tumour and adjacent tumour-free tissue was determined in defined areas (250 $\mu m^2$) by digital light measurement. These defined areas did not overlay blood vessels with a diameter $> 30\,\mu m$. The image analysis was used for an interactive frame positioning. Thus areas of measurement were identical for all time points. Spatial inhomogeneities of the light source and the camera were compensated by shading correction performed with the image analysis system.

*Chemical extraction*

After the final intravital microscopic measurement, animals were sacrificed by an overdose of anaesthesia (Pentobarbital, 500 mg $kg^{-1}$ b.w.) i.p. and tissue specimens of the tumour and adjacent tumour-free tissue were immediately excised and frozen in liquid nitrogen for chemical extraction.

To enable measurement of rapid serum kinetics for the three porphycenes (dose: 1.4 $\mu$mol $kg^{-1}$ b.w.), a two catheter blood sampling technique was applied to a second group of 21 hamsters (ATPPn $n = 8$, HEPn $n = 9$, CBPn $n = 4$). The minimum interval between injection and sampling was 30 s. Blood samples (40 $\mu$l) from anesthetised hamsters bearing a venous catheter as described above and an additional catheter implanted in the right carotid artery were taken in heparinised capillaries. The samples were centrifuged to determine the hematocrit and to isolate serum used for chemical extraction.

Serum and tissue specimens were extracted by a procedure that recovered 85 ± 5% of every porphycene employed from serum and erythrocytes, and extracted <10% of the initially extracted amount of dye from the tissue sediments in re-extraction experiments. Ten $\mu$l serum samples or weighed amounts of tumour and tumour-free tissue (5–20 mg), cut in small pieces, were treated with methanol. Serum probes were briefly agitated and tissue specimen homogenised in a Potter-Elvehjem vessel followed by subsequent additions of acetonitrile containing Diphenylanthracene (DPA) reference standard. Samples were sonicated, and centrifugation yielded a supernatant that was subjected to spectrofluorometric analysis. The efficiency of the extraction procedure was determined by comparing the fluorescence of extracts from porphycenes incubated either with erythrocytes, serum or buffer alone (control).

Emission spectra were recorded on a spectrofluorometer equipped with a red sensitive phototube at a 5 nm slidewidth. Light source intensity was calibrated against $10^{-7}$ M tetraphenylbutadiene solid standard in a polymethylmethacrylate matrix (Starna, Pfungstadt, Germany). Peak intensities were read against DPA internal standard intensities at 429 nm and compared to the porphycene calibration plots. ATPPn 72 h tissue extracts eventually exhibited an additional 670 ± 10 nm peak, showing porphycene excitation characteristics, whose intensity was quantified as the ATPPn 644 nm maximum.

During the sampling, some of the interstitial fluid was lost (<20%), which might have led to a slightly lower tumour selectivity of ATPPn (Table IV) compared to the microscopic measurements.

The extraction protocol was restricted to the porphycenes since no method for the quantitative extraction of Photofrin, whose chemical composition is still partly unknown (Pandey *et al.*, 1990) could be established.

*Mathematical analysis and statistics*

Least square fits, using a Marquardt algorithm (Bevington, 1969), were employed for the serum kinetics. Serum kinetics were analysed according to the two compartment model (Benet *et al.*, 1980). Eventual deviations of injected sensitiser dose were corrected, by factorising deviation from 1 min averages of individual serum concentrations. Loss of blood volume caused by repeated blood sampling from the animals was corrected for by adjusting serum concentrations to the actual hematocrit.

For nonparametric one-way analysis of variance and multiple comparison of ranks of several independent samples, the Kruskal-Wallis test was utilised. Single comparisons of unpaired samples were performed using U-test and of paired samples by the Wilcoxon-test. Data are given as mean ± standard deviation (SD) or standard error of the mean (SEM), respectively. Tumour:skin tissue fluorescence ratios (Figure 6) are means of ratios of individual animals, diverging slightly from the ratio of mean fluorescence of each tissue (Figure 5).

### Results

Pharmacokinetics of the porphycenes showed a strong dependence on the chemical properties of the sensitisers (Figure 1 and Figure 4). For each single porphyrinoid, time constants for uptake and elimination from melanomas were similar to those found for skin tissue and blood. The rates of accumulated or eliminated molecules, however, differed distinctly between the tissues monitored.

Halflife of distribution from serum decreased in the order ATPPn > HEPn = CBPn (Figure 3, and Table I). HEPn additionally exhibited a redistribution phase in its serum kinetics prolonging its full distribution and reflecting the HEPn > CBPn order in tissue uptake. Actual serum halflives of distribution were determined to be 7 h for the alkylporphycene ATPPn and 1 min for both etherporphycenes by the fit procedure (Table I). The redistribution process had a halflife of 1 h for HEPn (Table I). Elimination halflife also decreased in the order ATPPn > HEPn > CBPn, with values of more than 1 day for ATPPn, about half a day for HEPn, and 3 h for CBPn. Thus, the acceleration of the pharmacokinetics followed the polarity of the porphycenes.

One minute after injection, the most lipophilic alkylporphycene ATPPn had not penetrated erythrocytes effectively, whereas both trietherporphycenes were found in considerable amounts in red blood cells. At that time, dye concentration ratio of serum to red blood cells was 130:1 for ATPPn, 2.3:1 for HEPn, and 1.4:1 for CBPn (Table II). After 10 h, less than 10% of the serum concentration of ATPPn was found in erythrocytes.

Tissue uptake, defined as the time interval to reach maximum tissue fluorescence, was shortened in the order CBPn > HEPn > Pf > ATPPn. Maximum fluorescence intensity in tumours was detected 30 s after injection for CBPn, 3 h after injection of HEPn, and 24 h after injection of ATPPn (Figure 4). Tumour fluorescence reached a maximum 6 h after Pf injection and increased again after 24 h, though not significantly.

**Figure 3** Serum-kinetics of porphycenes. **a**, ATPPn; **b**, HEPn; **c**, CBPn. Serum-levels were spectrofluorometrically measured from extracted samples. Bars indicate typical errors, taken from the s.d. of repeated extraction of one sample in every concentration range. Lines give results of fit procedure. The insert is a bilogarythmic plot of identical values. Note the x-axis of the CBPn plot being 1/10 of ATPPn and HEPn plots.

**Table I** Serum distribution (D), redistribution (R), and elimination (E) halflives of the porphycenes

| *Porphycene* | $t_{\frac{1}{2}}D$ *(min)* | $t_{\frac{1}{2}}R$ *(min)* | $t_{\frac{1}{2}}E$ *(min)* |
|---|---|---|---|
| ATPPn | 430 ± 30 | – | 1700 ± 1300 |
| HEPn | 0.91 ± 0.1 | 62 ± 8 | 500 ± 160 |
| CBPn | 1.1 ± 0.3 | – | 170 ± 30 |

Values are derived from fits to serum-kinetics, determined by chemical extractions; errors are s.d.

**Table II** Porphycene concentration ratios serum to erythrocytes 1 min after i.v. injection

| *Sensitiser* | *Ratio*$_{C_{serum}/C_{ervs}}$ |
|---|---|
| ATPPn | 130:1 |
| HEPn | 2.3:1 |
| CBPn | 1.4:1 |

Fluorometric measurements in the tumour bearing chambers demonstrated that uptake and elimination in the skin tissue was coincident with uptake and elimination in the melanoma for all sensitiser tested, however, the amount of photosensitisers detected in both tissues differed distinctly. Both fluorescence and extraction measurements yielded this result independently (Figure 4, Table III and IV). Only two of the photosensitisers (ATPPn and Pf) were found to localise preferentially in tumours. ATPPn was delivered far more effectively to tumours than to non-neoplastic tissue. Photofrin also accumulated in the melanoma, though in a less pronounced way. HEPn only showed slight tumour selectivity 30 s after injection, but later reached higher concentrations in subcutaneous host tissue than in tumours. To our knowledge, HEPn is the first photosensitiser that localises 'anti-tumour-selectively'. The most polar porphycene, CBPn, showed no specificity for the fluorometrically monitored tissues.

The time courses in tumour specificity are given as the fluorescence ratio of tumour:skin tissue in Figure 5. These 'selectivity functions' showed a strong time dependence for ATPPn and HEPn and to a lesser degree for Photofrin. The maximum tumour selectivity ratio was 3.2 for ATPPn, 1.7 for Pf, and 1.3 for HEPn (Table IV). Increases in tumour selectivity occurred during the uptake phases of tissue kinetics for the tumour-selective sensitisers ATPPn, Pf, and HEPn. The halflife for the tumour selection process of ATPPn equalled the serum distribution constant within experimental error. The time constant for selectivity of HEPn was between the halflives for redistribution and elimination from the serum. No increase in tumour selectivity was seen during elimination periods from tissues and serum for any of the sensitisers tested and decreased for both the highly tissue specific porphycenes ATPPn and HEPn.

Fluorescence pictures taken of tumour chambers 24 h after dye injection (Figure 6) showed peak emission intensities at tumour edges for ATPPn. The anti-tumour selective porphycene, HEPn, was located in large, diffuse spots and stripes in the subcutaneous tissue and was not localised in tumours and/or the vasculature as evidenced by fluorometric measurements.

### Discussion

The elucidation of the tumour localisation of porphyrin(oid)s presents a challenge to PDT researchers, since understanding of the underlying mechanism(s) might allow the design of not only improved photodynamic, but also non-photodynamic drugs. Intravital microscopy of tumours grown in skin chambers has proved to be a valuable tool for the study of microcirculation (Endrich *et al.*, 1989; Asaishi *et al.*, 1981; Leunig *et al.*, 1992). It appears to be a suitable technique for the study of porphyrinoid localisation since these PDT drugs can be directly visualised by their fluorescence. Thus pharmacokinetics can be determined at the microscopic level without artifacts caused by sacrifice of the animal with high experimental accuracy, since the local pharmacokinetic can be measured from one individual. Additionally fast diffusional processes, not observable by *ex vivo* techniques, can be monitored. However, care must be taken to ensure a linear correlation between fluorescence intensity and drug concentration in the tissue, for well known aggregation and quenching phenomena can lead to a deviation from linearity. In the present study intravital fluorescence microscopy was compared to chemical extraction of microsurgically obtained tissue samples from monitored areas. The agreement of results within experimental errors demonstrates the validity of the approach (Table IV).

The results of the present investigation demonstrate that preferential localisation in tumours is not a feature common to all porphyrinoids. Among the three pure model compounds tested, only the most lipophilic dye, ATPPn, showed a strong positive affinity for the amelanotic melanoma (A-Mel-3). The tissue kinetics of this tumour selective porphycene (ATPPn) and Photofrin appear strikingly similar (Figure 4a and d). Both sensitisers show the increase in tumour-selectivity (Figure 5a and b) during the uptake phase into tissues. For ATPPn the uptake nature of the tumour localisation is additionally demonstrated by the similarity of time constants for the rise in tumour-specificity (260 ± 150 min) and the distribution from the serum (430 ± 30 min). The prolonged uptake of Photofrin compared to ATPPn can be rationalised in terms of the long persistence of a distinct hydrophobic fraction of the porphyrin mixture in the serum (Bellnier *et al.*, 1989).

The observed accumulation of ATPPn and Photofrin during their delivery from the bloodstream strongly suggests that uptake and not retention-type tumour-localisation mechanisms lead to the tumour preferential localisation of porphyrines. If compromised lymphatic drainage (Gullino, 1975) and/or prolonged retention of protogenic molecules in low pH tissues (Brault *et al.*, 1986; Barret *et al.*, 1990) were the underlying localisation mechanisms, an increase of the tumour selectivity should appear during the elimination phases of tissue kinetics. The decrease in tumour:skin tissue ratio during the elimination of HEPn and ATPPn (Figure 5a) points to the absence of a specific dye retention in the tumour model used in this study.

From our results, a model can be proposed to explain the localisation kinetics of Photofrin. Assuming that the Photofrin mixture contains a fraction of anti-tumour selective molecules like HEPn besides an ATPPn-like localising fraction its lower selectivity can be explained. This assumption seems reasonable because the dihydroxyethylporphyrine hematoporphyrine, is known to be present in Photofrin (Dougherty *et al.*, 1987). Since the time constant for the process leading to the anti-tumour selectivity of HEPn is similar to the time constant of the positive process of ATPPn (Table III), the two Photofrin fractions corresponding to these porphycenes might localise coincidently in opposite 'directions'. Thus, the weak time dependence in the tumour-selectivity of Photofrin (Figure 5b) is understandable.

Photofrin is known to be lipophilic (Dougherty *et al.*, 1983; Kessel & Chou, 1983; Dougherty, 1987) and ATPPn is the most lipophilic sensitiser among the porphycenes tested. ATPPn remains within lipoprotein serum carrier particles for hydrophobic molecules or residual liposomes as indicated by its low concentration in erythrocytes (Table II). This finding is in accordance with earlier studies with a similar porphycene (Guardiano *et al.*, 1989). The porphyrins in photofrin will probably form aggregates in watery solutions or, upon injection, become associated with serum proteins (Cohen & Margalit, 1990). The negligible uptake of ATPPn

**Figure 4** Tissue fluorescence-kinetics in the melanoma and skin tissue. **a**, ATPPn; **b**, HEPn; **c**, CBPn; **d**, Pf. Closed circles indicate tumour, open circles tumor-free subcutaneous tissue. Fluorescence intensities of selected areas in tumour and tumour-free tissue are given relative to solid fluorescence reference signals as mean ± s.e.m. Tumour fluorescence was significantly higher compared to the tumour-free subcutaneous tissue for ATPPn and Pf ($P<0.001$), not significantly different for CBPn, and significantly lower for HEPn ($P<0.001$). Note the x-axis of the CBPn plot being 1/3 of other plots.

**Figure 5** Tumour to skin tissue ratios. **a**, ATPPn and HEPn; **b**, CBPn and Photofrin. Tumour to tumour-free tissue ratios in fluorescence intensities of monitored areas as determined by intravital microscopy. Values are mean of the ratio of individual animals ± s.e.m.

**Table III** Time to reach half maximum ($t_{½}$) and maximum ($t_{max}$) tumour selectivity ratio

| *Sensitizer* | $t_{½}$ *(min)** | $t_{max}$ *(h)*† |
|---|---|---|
| Pf | (a) | 1 |
| ATPPn | 260 ± 150 | 24 |
| HEPn | 170 ± 30(b) | 0.01 |
| CBPn | (c) | 6 |

Values are mean ± s.d. (a) Not unambiguously evaluable, (b) process leading to skin tissue selective localisation, and (c) no time dependence. *Determined by a monoexponential fit process to data of Figure 5. †Directly obtained from acquired data.

in skin tissue furthermore demonstrates that there is no fast membrane bound mechanism leading to the uptake of this sensitiser. The tissue uptake of ATPPn and Photofrin might, therefore, be governed by their macromolecular carrier particles rather than by the molecules themselves. Interstitial concentrations of IgG in tumours after a bolus injection as reported by Jain & Baxter (1988) showed a similar time course of accumulation compared to ATPPn and Photofrin further supporting a delivery of these photosensitisers via serum macromolecules.

The macromolecules can accumulate in tumours either by endocytotic processes or by extravasating from the blood through holes in the endothelial lining. Both an enhanced number of LDL receptors on tumour cells (Gal *et al.*, 1981; Norata *et al.*, 1985; Maziere *et al.*, 1991) and high vascular permeability of tumours for macromolecules (Jain, 1987) are known. If the endocytotic activity of the tumour cells was the main accumulation mechanism, the interstitial fluid of tumours should be impoverished in sensitiser compared to the surrounding tissue and tumour cells should show bright fluorescence. The microscopic pictures revealed, however, strongly fluorescent tumour edge edema in (Figure 6b) as expected for vascular permeability as major reason for the tumour localisation.

The permeability hypothesis might also explain the selectivity found for a wide range of different photosensitisers (Gomer, 1991), many of which were shown to have an affinity to macromolecular carrier particles in the bloodstream, like lipoproteins or albumin (Cohen *et al.*, 1990). In accordance with this 'vascular' localisation process, no difference in sensitiser uptake had been found between malignant and non-malignant cell lines *in vitro* (Moan *et al.*, 1981).

**Figure 6** Photomicrographs of the amelanotic melanoma. **a**, Trans-illumination, photomicrograph of an A-Mel-3 tumour (diameter: 4 mm) localised at the left lower edge of the chamber preparation, 6 days after implantation. **b**, Epi-illumination 24 h subsequent to i.v. injection of 1.4 $\mu$mol $kg^{-1}$ b.w. ATPPn as recorded by intravital microscopy. Note the strong fluorescence within the tumour surrounding edema. **c**, Epi-illumination of a tumour chamber 24 h after i.v. injection of 1.4 $\mu$mol $kg^{-1}$ b.w. HEPn. Note the non fluorescent tumour and tumour surrounding edema. The two fluorescent squares at the top of the chamber represent the reference signals. (The bars represent 2 mm).

**Table IV (a)** Tumour selectivity ratio at 6 h ($r_6$) and 72 h ($r_{72}$), and maximum tumour selectivity ratio ($r_{max}$) as evaluated by fluorescence microscopy

| *Sensitiser* | $r_6$ *(h)* | $r_{72}$ *(h)* | $r_{max}$ |
|---|---|---|---|
| Pf | 1.6 ± 0.2 | 1.7 ± 0.4 | 1.7 ± 0.3 |
| ATPPn | 2.3 ± 0.7 | 2.7 ± 1.3 | 3.2 ± 1.1 |
| HEPn | 0.6 ± 0.2 | 0.3 ± 0.1 | 1.3 ± 0.2 |
| CBPn | 1.1 ± 0.3 | – | 1.1 ± 0.3 |

Values are mean ± s.d.

**(b)** Tumour selectivity ratio at 6 h ($r_6$) and 72 h ($r_{72}$) as evaluated by chemical extraction

| *Sensitiser* | $r_6$ *(h)* | $r_{72}$ *(h)* |
|---|---|---|
| Pf | – | – |
| ATPPn | 1.8 ± 0.4* | 2.0 ± 0.6 |
| HEPn | 0.64 ± 0.1* | 0.4 ± 0.2 |
| CBPn | 1.1 ± 0.2* | – |

Values are mean ± s.d. except for those marked *, these are mean ± error of two extractions.

Further evidence for the 'selective macromolecule filtration assumption of macromolecules comes from the localisation of the non-tumour selective porphycenes HEPn and CBPn. Unlike macromolecular bound molecules (Nugent & Jain, 1984), HEPn and CBPn are fast distributed from the blood (Table I) as seen for small molecules (Gerlowski & Jain, 1983; Gibaldi & Perrier, 1982) and penetrate erythrocytes effectively (Table II). Small standard deviations of the tissue distributions (Figure 4b and c) support distribution processes driven by physical gradients for small molecules.

The almost instantaneous tissue uptake of CBPn, which reached its maximum 30 s after the end of dye injection favours free diffusion as possible transport mechanism. The distribution of the less polar hydroxyporphycene HEPn probably proceeds via fast membrane uptake as it has been observed for the hydroxysteroid cholesterol (Barclay *et al.*, 1990). HEPn reaches its maximum tissue concentrations relatively slowly, probably via diffusion along hydrophobic structures. The equilibrium distribution pattern seems to be governed by the amount of hydrophobic sites within a tissue. Highly fluorescent fat cells found in the subcutaneous host tissue at high magnification and the high water content of the amelanotic melanoma* support this explanation. Hence, the pharmacokinetics of HEPn do not seem to favour earlier assumptions of a general accumulation of lipophilic molecules by sensitiser retaining tissues (Freitas, 1990).

In conclusion, our study demonstrates that intravital microscopy is a valuable tool for the study of porphyrinoid pharmacokinetics. Investigating synthetic porphyrinoids of variable lipophilicity, it was shown that polar functional groups accelerate the pharmacokinetics of photosensitisers. The most lipophilic porphycene studied accumulated in tumours twice as much as Photofrin. Local tumour selectivity of sensitisers was found to originate in uptake and not elimination processes in the body compartment monitored. Increased permeability of the tumour vasculature to carrier macromolecules probably plays a governing role in the localisation process. Extension of these experiments to other physiological compartments, tumour models and photosensitisers will be needed to support these conclusions.

Porphycenes were synthesised by M. Müller, M. Kisters, T. Benninghaus, and C. Richert, at the Institute for Organic Chemistry, University of Cologne. The authors gratefully acknowledge the skillful assistance of Drs P. Heil, W. Beyer, W. Müller and G. Kuhnle for computer programs, the GSF Research Center, Zentrales Laserlaboratorium (E. Unsöld) for providing laboratory facilities, Dr J. Wessel for time resolved fluorescence measurements and Drs R. Baumgartner, L.T. Baxter, L.E. Gerweck, R.K. Jain, E.N. Kaufman, K. Messmer, and T.C. Zankel for critical comments on the manuscript.

This work was supported by the Bundesministerium für Forschung und Technologie to A.E. Goetz (No. 0706903A5). M. Leunig is a recipient of a Feodor-Lynen Fellowship from the Humboldt Foundation, and C. Richert is a recipient of a Hanns Seidel predoctoral Fellowship.

**Abbreviations**

ATPPn, Acetoxytetrapropylporphycene: CBPn, Carboxymethoxycarbonylbenzotris(methoxyethyl)porphycene; DPA, Diphenylanthracene; DOPC, Dioleylphospharidylcholine; HEPn, Hydroxyethyltris(methoxyethyl)porphycene; n, Number of animals; PBS, Phosphate buffered saline; PDT, Photodynamic Therapy; Pf, Photofrin; SIT, Silicon intensified target.

### References

ARAMENDIA, P.F., REDMOND, R.W., NONELL, S., SCHUSTER, W., BRASLAVSKY, S.E., SCHAFFNER, K. & VOGEL, E. (1986). The photophysical properties of porphycenes: Potential photodynamic therapy agents. *Photochem. Photobiol.*, **44**, 555–559.

ASAISHI, K., ENDRICH, B., GOETZ, A. & MESSMER, K. (1981). Quantitative analysis of microvascular structure and function in the amelanotic melanoma A-Mel-3. *Cancer Res.*, **41**, 1898–1904.

AULER, H. & BANZER, G. (1942). Untersuchungen über die Rolle der Porphyrine bei geschwulstkranken Menschen und Tieren. *Z. Krebsforsch.*, **53**, 65–68.

BARCLAY, L.R.C., CAMERON, R.C., FORREST, B.J., LOCKE, S.J., NIGAM, R. & VINQUIST, M.R. (1990). Cholesterol: free radical peroxidation and transfer into phospholipid membranes. *Biochem. Biophy. Acta.*, **1047**, 255–263.

BARRET, A.J., KENNEDY, J.C., JONES, R.A., NADEAU, P. & POTTIER, R.H. (1990). The effect of tissue and cellular pH on the selective biodistribution of porphyrin-type photochemotherapeutic agents: a volumetric titration study. *J. Photochem. Photobiol. B: Biology*, **6**, 309–323.

BAUMGARTNER, R., FISSLINGER, H., JOCHAM, D., LENZ, H., RUPRECHT, L., STEPP, H. & UNSÖLD, E. (1987). A fluorescence imaging device for endoscopic detection of early stage cancer -instrumental and experimental studies. *Photochem. Photobiol.*, **46**, 759–763.

BELLNIER, D.A., HO, Y.K., PANDEY, R.K., MISSERT, J.R. & DOUGHERTY, T.J. (1989). Distribution and elimination of photofrin II in mice. *Photochem. Photobiol.*, **50**, 221–228.

BENET, L.Z. & SHREINER, L.B. (1980). Pharmacokinetics: The dynamics of drug absorption, distribution and elimination. In *The Pharmacological Basis of Therapeutics*, Goodman & Gilman's, (eds) pp. 3–34. Macmillan: New York.

BEVINGTON, R.D. (1969). *Data Reduction and Error Analysis for the Physical Sciences.* McGraw Hill: New York.

BRAULT, D., VEVER-BIZET, C. & LEDOAN, T. (1986). Spectrofluorimetric study of porphyrin incorporation into membrane models – evidence for pH effects. *Biochim. Biophys. Acta.*, **857**, 238–250.

COHEN, S. & MARGALIT, R. (1990). Binding of porphyrin to human serum albumin. *Biochem J.*, **270**, 325–330.

DELLIAN, M., RICHERT, C., GAMARRA, F. & GOETZ, A.E. (1992). Tumor growth following PDT with functionalized prophycenes and photofrin II. In *Photodynamic Therapy and Biomedical Lasers*, Spinelli, P., Dal Fante, M., & Marchesini, R. (eds) pp. 467–469. Elsevier Science Publishers B.V.: Amsterdam.

DOUGHERTY, T.J. (1987). Studies on the structure of porphyrins contained in photofrin II. *Photochem. Photobiol.*, **46**, 569–573.

DOUGHERTY, T.J., BOYLE, D.G., WEISHAUPT, K.R., HENDERSON, B.A., POTTER, W.R., BELLNIER, D.A. & WITYK, K.E. (1983). Photoradiation therapy -clinical and drug advances. In *Porphyrin Photosensitization*, Kessel, D. & Dougherty, T.J. (eds) pp. 3–13. Plenum Press: New York.

DOUGHERTY, T.J., KAUFMAN, J.E., GOLDFARB, A., WEISHAUPT, K.R., BOYLE, D. & MITTLEMAN, A. (1978). Photoradiation therapy for the treatment of malignant tumors. *Cancer Res.*, **38**, 2628–2635.

ENDRICH, B., HAMMERSEN, F., GOETZ, A. & MESSMER, K. (1982). Microcirculatory bloodflow, capillary morphology, and local oxygen pressure of the hamster amelanotic melanoma A-Mel-3. *J. Natl Cancer Inst.*, **68**, 475–485.

ENDRICH, B., ASAISHI, K., GOETZ, A. & MESSMER, K. (1980). Technical report. A new chamber technique for microvascular studies in unanaethesized hamsters. *Res. Exp. Med.*, **177**, 125–134.

FREITAS, L. (1990). Lipid accumulation. The common feature to photosensitizer-retaining normal and malignant tissues. *J. Photochem. Photobiol. B: Biology*, **7**, 359–361.

GAL, D., MCDONALD, P.C., PORTER, J.C. & SIMPSON, E.R. (1981). Cholesterol metabolism in cancer cells in monolayer culture. III. Low density lipoprotein metabolism. *Int. J. Cancer*, **28**, 315–319.

GERLOWSKI, L.E. & JAIN, R.K. (1983). Physiologically based pharmacokinetic modeling: principals and applications. *J. Pharm. Sci.*, **72**, 1103–1123.

GIBALDI, M. & PERRIER, D. (1982). *Pharmacokinetics*, M. Dekker, Inc.: New York.

GOMER, C.J. (1991). Preclinical examination of first and second class generation photosensitizers used in photodynamic therapy. *Photochem. Photobiol.*, **54**, 1093–1107.

GUARDIANO, M., BIOLO, R., JORI, G. & SCHAFFNER, K. (1989). Tetra-n-propylporphycene as a tumour localizer: pharmacokinetic and phototherapeutic studies in mice. *Cancer Lett.*, **44**, 1–6.

GULLINO, P.M. (1975). Extracellular compartments of solid tumors. In *Cancer Vol 3*, Becker (ed.) pp. 327–354. Plenum Press: New York.

JAIN, R.K. (1987). Transport of macromolecules across tumor vasculature. *Cancer Metast. Rev.*, **6**, 559–594.

JAIN, R.K. & BAXTER, L.T. (1988). Mechanisms of heterogeneous distribution of monoclonal antibodies and other macromolecules in tumors: significance of elevated interstitial pressure. *Cancer Res.*, **48**, 7022–7032.

KESSEL, D. & CHOU, T. (1983). Tumor localizing components of the porphyrin preparation hematoporphyrin derivative. *Cancer Res.*, **43**, 1994–1999.

KREIMER-BIRNBAUM, M. (1989). Modified porphyrins, chlorins, phthalocyanines and purpurines: Second generation photosensitizers for photodynamic therapy. *Semin. Hematol.*, **26**, 157–173.

LEUNIG, M., YUAN, F., MENGER, M.D., BOUCHER, Y., GOETZ, A.E., MESSMER, K. & JAIN, R.K. (1992). Angiogenesis, microvascular architecture, microhemodynamics, and interstitial fluid pressure during early growth of human adenocarcinoma LS174T in scid mice. *Cancer Res.*, **52**, 6553–6560.

LIPSON, R.L., BALDES, E.J. & OLSEN, A.M. (1961*a*). The use of a derivative of hematoporphyrin in tumor detection. *J. Natl Cancer Inst.*, **26**, 1-11.

LIPSON, R.L., BALDES, E.J. & OLSEN, A.M. (1961*b*). Hematoporphyrin derivative: A new aid for endoscopic detection of malignant disease. *J. Thorac. Cardiov. Sur.*, **42**, 623-629.

MAZIERE, J.C., MOLIERE, P. & SANTUS, R. (1991). The role of low density lipoprotein receptor pathway in the delivery of lipophilic photosensitizers in the photodynamic therapy of tumours. *J. Photochem. Photobiol. B: Biology*, **8**, 351-360.

MEYER-BETZ, F. (1913). Untersuchungen über die biologische (photodynamische) Wirkung des Hämatoporphyrins und anderer Derivate des Blut- und Gallenfarbstoffes. *Dtsch. Arch. Klin. Med.*, **112**, 476-503.

MOAN, J. & BERG, K. (1992). Photochemotherapy of cancer: experimental research. *Photochem. Photobiol.*, **55**, 931-948.

MOAN, J., STEEN, H.D., FEREN, K. & CHRISTENSEN, T. (1981). Uptake of hematoporphyrine derivative and sensitized photoinactivation of CH3 cells with different oncogenic potential. *Cancer Lett.*, **14**, 291-296.

NORATA, G., CANTI, L., RICCI, L., NICOLIN, A., TREZZI, E. & CATAPANO, A.L. (1985). *In vivo* assimilation of low density lipoproteins by a fibrosarcoma tumour line in mice. *Cancer Lett.*, **25**, 203.

NUGENT, L.J. & JAIN, R.K. (1984). Plasma pharmacokinetics and interstitial diffusion of macromolecules, *Am. J. Physiol.*, **246**, H129-H137.

PANDEY, R.K., SIEGEL, M.M., TSAO, R., MCREYNOLDS, J.H. & DOUGHERTY, T.J. (1990). Fast atom bombardment mass spectral analysis of photofrin II and its synthetic analogs. *Biomed. Environ. Mass. Spectrom.*, **19**, 405-414.

POLICARD, A. (1924). Etudes sur les aspects offerts par des tumeurs experimentales examinees a la lumiere de Wood. *CR. Soc. Biol.*, **91**, 1423-1424.

RICHERT, C. (in press). A long-time stable liposome formulation for porphyrinoid photosensitizers. *J. Photochem. Photobiol. B: Biology.*

SYNDER, L.R. (1974). Classification of the solvent properties of common liquids. *J. Chromatogr.*, **92**, 223-230.

VOGEL, E. (1990). Novel porphyrinoids. *Pure & Appl. Chem.*, **62**, 557-564.

VOGEL, E., BALCI, M., PRAMOD, K., KOCH, P., LEX, J. & ERMER, O. (1987). 2,7,12,17-Tetrapropylporphycene -counterpart of octaethylporphyrin in the porphycene series. *Angew. Chem. Int. Ed. Engl.* **26**, 909-912; *Angew. Chem.*, **99**, 909-912.

VOGEL, E., KÖCHER, M., SCHMICKLER, H. & LEX, J. (1986). Porphycene -a novel porphin isomer. *Angew. Chem. Int. Ed. Engl.*, **25**, 257-259; *Angew. Chem.*, **98**, 262-264.